# Increased Levels of Granulocytic Myeloid-Derived Suppressor Cells in Peripheral Blood and Tumour Tissue of Pancreatic Cancer Patients

**DOI:** 10.1155/2014/879897

**Published:** 2014-01-29

**Authors:** Yazan S. Khaled, Basil J. Ammori, Eyad Elkord

**Affiliations:** ^1^Institutes of Cancer, Inflammation & Repair, University of Manchester, Manchester M20 4BX, UK; ^2^Biomedical Research Centre, School of Environment & Life Sciences, University of Salford, The Crescent, Peel Building, Manchester M5 4WT, UK; ^3^Department of Upper Gastrointestinal Surgery, Salford Royal NHS Foundation Trust, Manchester M6 8HD, UK; ^4^Department of Hepatobiliary Surgery, North Manchester General Hospital, Manchester M8 5RB, UK; ^5^Section of Translational Anaesthetic and Surgical Sciences, Leeds Institute of Molecular Medicine, Leeds LS9 7TF, UK; ^6^College of Medicine & Health Sciences, United Arab Emirates University, P.O. Box 17666, Al Ain, UAE

## Abstract

Pancreatic cancer (PC) often presents late with poor survival. While role of immunosuppressive cells in preclinical studies provided help to develop immunotherapeutic agents, these cells remain under investigation in PC. The aim of this study was to characterise the different subsets of myeloid-derived suppressor cells (MDSCs) and evaluate their level and function in the circulation and tissue of PC patients. Significant increases in circulating and tumour-infiltrating granulocytic (Lin-HLA-DR-CD33+CD11b+CD15+), but not monocytic (Lin-HLA-DR-CD14+), MDSCs were detected in PC patients when compared with healthy donors and patients with chronic pancreatitis. The circulating MDSCs from PC patients expressed arginase 1, which represents their functional state. Blood levels of MDSCs showed no association with PC stage or preoperative levels of tumour markers. These findings provide a first characterisation of the phenotype of different subsets of peripheral and local MDSCs in PC patients and suggest that the frequency and contribution of these cells are predominantly granulocytic. This information demonstrates that MDSCs play a role in pancreatic cancer and future large validation studies may help in the development of new immunotherapeutic strategies to inhibit or eliminate MDSC function.

## 1. Introduction

Pancreatic adenocarcinoma (PAC) affects 1 in 10,000 populations and carries a poor prognosis [1]. The disease often presents with an advanced stage, and only 10–15% of patients are suitable for surgical resection [2]. The 5-year survival rate for patients with operable disease is estimated to be 15–40% [3]. The relationship between chronic inflammation and cancer has been recognised for decades, and at least 15–20% of cancers are associated with inflammation [4]. Chronic pancreatitis (CP) predisposes to pancreatic carcinoma [5]. Tumour-induced immunosuppression is widely accepted as a key mechanism by which tumours evade the immune system. It is now evident that immune responses in cancer are negatively regulated by immunosuppressive cells, mainly T regulatory cells (Tregs) and myeloid-derived suppressor cells (MDSCs). Recent research has demonstrated that the expansion and accumulation of MDSCs constitute one of the important mechanisms of tumour immune evasion. Treg levels are increased in pancreatic adenocarcinoma (PAC) and their high levels are associated with poor prognosis and reduced survival [6]. In mice with spontaneous pancreatic carcinoma, an increase in the levels of MDSC early in tumour development was detected in lymph nodes, blood, and pancreata of mice with premalignant lesions and increased further upon tumour progression [7]. They are largely responsible for inhibiting host T-cell activity against tumour-associated antigens (TAAs) and consequently impair the effectiveness of anticancer immunotherapeutic approaches [8]. Therefore, reducing the deleterious effects of these immunosuppressive cells may increase the success of various immunotherapeutic modalities in cancer [9]. However, the data on the role of MDSCs in patients with pancreatic cancer (PC) are limited. Therefore, characterisation of these immunosuppressive cells could have important implications on assessment of the prognosis and the exploration of therapeutic approaches for this challenging cancer. In addition, MDSC accumulation has been associated with the progression of cancer with some evidence that their elimination can enhance cancer immunotherapy+ [10].

MDSCs are defined as a heterogeneous population of activated immature myeloid cells characterised by a morphological mixture of granulocytic and monocytic cells, but they lack the expression of cell-surface markers that are specific to the fully differentiated monocytes, macrophages, or dendritic cells [11]. In mice, granulocytic MDSCs have a CD11b^+^Ly6G^+^Ly6C^low^ phenotype, whereas MDSCs with monocytic morphology are CD11b^+^Ly6G^−^Ly6C^high^ [11–15]; both phenotypes have different functions in cancer and autoimmune diseases [12, 16]. In contrast, the human MDSCs are traditionally defined as CD14^−^CD11b^+^CD33^+^CD15^+^ cells or cells that express the CD33 marker but lack the expression of markers of mature myeloid and lymphoid cells and the major histocompatibility complex (MHC) class-II molecule HLA-DR [17–19]. The identification and isolation of human MDSC subsets have been challenging due to the heterogeneous characteristics of these immature cells and accumulating data suggest a significant diversity in the MDSC subsets identified in different human cancers. The frequency of each MDSCs subset seems to be influenced by the cancer type. Patients with renal, colon and lung cancers have increased levels of granulocytic MDSCs in the circulation [20–22], whereas monocytic MDSCs are increased in the blood of patients with melanoma, prostate cancer, hepatocellular carcinoma, or head and neck cancer [21, 23–25]. However, there remains a lack of comprehensive clinicopathological correlations between tumour progression and MDSC phenotypes and levels in tumour tissue and peripheral blood.

In the present study, we characterised the phenotype of circulating and tumour-infiltrating MDSCs and set a comparative analysis across pancreatic cancer and chronic pancreatitis patients and healthy individuals. We also evaluated the functional status of the MDSC subpopulations using arginase 1 activity and the clinical significance of MDSCs in pancreatic disease progression. This characterisation of the different MDSC subsets in PC provides the impetus to develop pharmacological strategies to alter the function of MDSCs in order to enhance the efficacy of PC treatment.

## 2. Material and Methods

### 2.1. Patients and Healthy Donors

Peripheral blood samples were collected from PC patients (*n* = 24) and CP patients (*n* = 12). Sixteen age-matched healthy donors were used as controls. Tumour (*n* = 7) and benign (*n* = 7) pancreatic tissue samples were collected from patients who underwent surgery at the North Manchester General Hospital, UK, from April 2012 to January 2013. Written consents were obtained from all patients before blood/tissue sampling on a research protocol approved by the national research ethical committee and the local research and development (R&D) department.  Table 1 shows the characteristic features of all patients in this study. All patients were diagnosed with PC for the first time and had not been previously treated. The levels of tumour biomarkers were also collected for CP and for PC patients before the first dose of chemotherapy or surgery for clinical correlation with the levels of MDSCs.

### 2.2. Whole Blood Staining and Cell Isolation

Blood samples were collected in a 50 mL Falcon tube (BD biosciences, UK) containing 200 *μ*L (1000 IU/mL) heparin. The samples were transported immediately to the laboratory to be processed. Two hundred microliter (*μ*L) of whole blood was taken for MDSC analysis divided into 100 *μ*L for nonstained tube and 100 *μ*L for stained tube with mouse anti-human monoclonal antibodies (mAb) for different MDSC markers, as explained below. Sample tubes were vortexed well and incubated for 25 minutes at 4°C. After the incubation, lysis buffer (BD FACS lysing solution) was added to lyse the red blood cells (RBCs) and then incubated for 15 minutes at room temperature. The samples were washed twice with phosphate buffered saline (PBS). The pellet was resuspended in 300 *μ*L of flow cytometry buffer (FCB) before analysis. Blood samples were processed to isolate peripheral blood mononuclear cells (PBMCs) for other investigations. Tissue samples were collected into a sterile tube containing Roswell Park Memorial Institute (RPMI-) 1640 and kept in ice until reached the laboratory to be processed immediately. The method used to digest the tumour samples and to analyse the tumour-infiltrating MDSCs was chosen by comparing the frequency of MDSC subsets for two tumour samples with the different methods (data not shown). The suspension of the minced tumour in 1 mg/ml collagenase (Sigma-Aldrich, UK), 100 ug/mL of hyaluronidase type V (Sigma-Aldrich, UK), and 30 IU/mL of DNase type I (Sigma-Aldrich, UK) and incubation on roller mixer overnight at room temperature were chosen to study tumour-infiltrating MDSCs. Briefly, tissue specimens from patients with PC and CP were minced under aseptic techniques into 2–4 mm pieces, resuspended in enzymatic cocktail, and incubated on roller mixer overnight at room temperature. Then, the cell suspension was passed through a Falcon 100 *μ*m cell strainer (BD Biosciences, Oxford, UK) to remove any large aggregates and debris. The suspension was washed twice with RPMI-1640 and centrifuged at 300 g for 5 minutes before counting and staining.

### 2.3. Antibodies and Flow Cytometric Analysis

Flow cytometric analysis was performed simultaneously on fresh whole blood and tissue suspension following enzymatic disaggregation. MDSC subsets were determined using the following antibodies: anti-Lin-FITC, anti-CD11b-APC-Cy7, anti-CD14-PerCP-Cy5.5, anti-CD15-PE-Cy7, anti-CD33-APC, and anti-HLA-DR-PE. All the mAbs were purchased from BD Biosciences. Six-colour flow cytometric analysis was performed on a BD FACSVerse flow cytometer. Analysis of the flow cytometric data was performed using the BD FACSuite software.

### 2.4. Functional Characterisation of MDSCs

Arginase activity was determined using arginase 1 mAbs from R&D systems (Oxford, UK). Intracellular staining was performed on 100 *μ*L of fresh whole blood with sheep anti-human/mouse arginase 1 fluorescein-conjugated polyclonal antibody. Anti-Lin-FITC was not used when arginase activity was examined; we checked and did not see any difference in the subpopulations defined whether we include the anti-Lin antibody or exclude it. We first added antibodies for surface markers (CD11b-APC-Cy7, CD14-PerCP-Cy5.5, CD15-PE-Cy7, CD33-APC and HLA-DR-PE) to the whole blood sample and then vortexed the sample tubes well and incubated them for 25 minutes at 4°C. RBCs were then lysed using BD FACS lysing solution. The samples were washed twice with PBS and the pellet was resuspended in 1 mL of freshly prepared eBioscience fixation/permeabilisation working solution. Tubes were then incubated for 45 minutes at 4°C in the dark. The samples were washed twice with permeabilisation buffer. Arginase 1 mAb was then added to the cell suspension and incubated in the dark for 30 minutes and then washed twice with permeabilisation buffer. The pellet was resuspended in 300 *μ*L of FCB for analysis.

### 2.5. Statistical Analysis

Comparisons of the frequencies of the following subsets in the study groups were performed: Lin-HLA-DR-, Lin-HLA-DR-CD33+11b+, Lin-HLA-DR-CD33+11b+CD15+, Lin-HLA-DR-CD14+, HLA-DR-/^low^CD14+, and HLA-DR-/^Low^CD33+. Statistical analysis was performed on GraphPad Prism 5.0 software (GraphPad Software, USA). Unpaired Student's *t*-test (Mann-Whitney test) was used to assess the differences between the study groups. Nonparametric Spearman test was used to assess the correlation between circulating MDSCs and cancer clinical stage. *P* value ≤ 0.05 was considered statistically significant. The data are presented as medians (range).

## 3. Results

### 3.1. Frequency of Granulocytic MDSCs Is Significantly Elevated in the Peripheral Blood of Patients with PC

**Figure 1:**
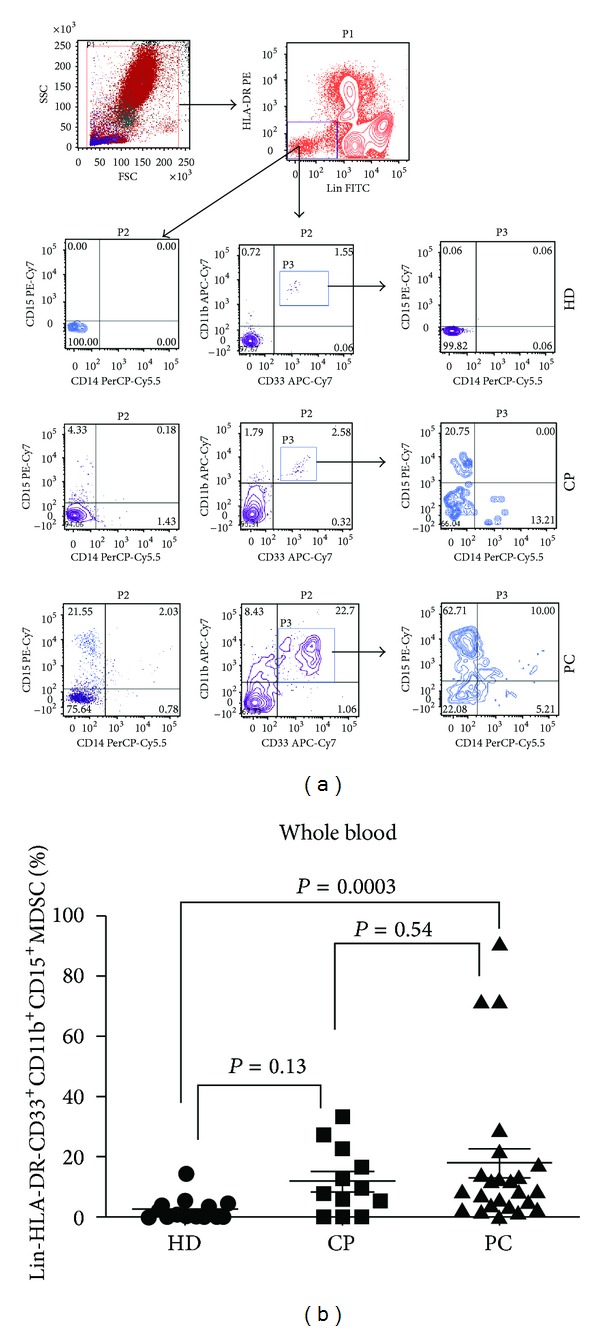
Levels of circulating MDSCs in patients with pancreatic cancer, compared to chronic pancreatitis patients and healthy donors. (a) Flow cytometric evaluation of Lin, CD33, CD11b, CD15, and CD14 in whole blood. An example of representative dot plots is shown for each study subgroup. Gates were set based on negative controls. Numbers represent the percentages from the original populations gated. P (number) above each FACS plot indicates the population gated that was analysed. The axis of each FACS plot represents the marker analysed. (b) Scatter plot of the percentage of Lin-HLA-DR-CD33+CD11b+CD15+ in the blood of the study groups. Bar represents median in each group. HD: healthy donor; CP: chronic pancreatitis; PC: pancreatic cancer.

Previous reports have described circulating MDSCs in human cancers as monocytic (HLA-DR-CD14+) [26] or granulocytic (CD14-CD15+) [27]. We aimed to study all the subsets of circulating MDSCs using multicolour flow cytometric analysis of whole blood from patients with PC, CP, and HDs using all the previously mentioned markers. We defined granulocytic MDSC as Lin-HLA-DR-CD33+CD11b+CD15+ and monocytic MDSC as Lin-HLA-DR-CD14+. Representative Flow Cytometric data of a normal HD, one patient with CP, and one with PC are shown in Figure 1(a). The frequency of circulating Lin-HLA-DR-CD33+CD11b+CD15+ subset was significantly higher in PC patients compared with HDs (8.86% versus 1.33%; *P* = 0.0003) but was not statistically higher in comparison with CPs (*P* = 0.54) as shown in Figure 1(b). In addition, there was no statistical difference in the levels of the Lin-HLA-DR-CD33+CD11b+CD15+ between HDs and CP patients. The frequency of Lin-HLA-DR-CD33+CD11b+CD15+ granulocytic subset was greater than monocytic Lin-HLA-DR-CD14+ in the peripheral blood of patients with PC (8.86% versus 0.89%, *P* = 0.004) and in those with CP (8.95% versus 1.35%, *P* = 0.003). However, we found no statistical difference in the frequency of circulating Lin-HLA-DR-CD14+ subset when comparing the three study groups. A more detailed description of the frequency of different MDSC subsets in peripheral blood of HDs, CP, and PC patients is shown in Table 2. Of note, the peripheral blood levels of monocytic MDSCs (HLA-DR-CD14+) were lower in the blood of PC (0.8%) group in comparison to HDs (1.3%) and CP (1.6%) groups, although this difference did not reach statistical significance.

### 3.2. Arginase 1 (ARG1) Expression in the Circulating MDSCs

**Figure 2:**
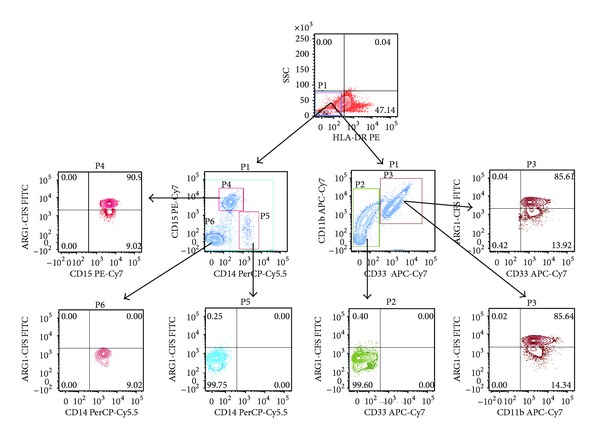
Arginase 1 expression in different subsets of circulating MDSC in patients with PC. Flow cytometric evaluation of ARG1 expression in CD33, CD11b, CD15 and CD14 in whole blood is shown in the representative dot plots. Gates were set based on negative controls. Numbers represent the percentages from the original populations gated. P (number) above each FACS plot indicates the population gated which was analysed. The gate was first set on HLA-DR negative against side scatter as shown in the top dot plot. Next, the CD11b+ & CD33+ (right) and CD14+ & CD15+ (left) subsets were identified. The expression of ARG1 in each subset was then determined as shown in the bottom dot plots.

High levels of ARG1 expression by MDSCs can accelerate the depletion of L-arginine in the tumour microenvironment, which subsequently inhibits T-cell proliferation by causing low expression of T-cell receptors and thus suppression of the cell cycle in T cells [28]. Others demonstrated that ARG1-producing MDSCs are granulocytic and they are increased in the circulation of human cancers [20, 29]. To investigate whether the circulating MDSCs express ARG1 in PC, flow cytometric based assay for ARG1 expression was used. We first gated on HLA-DR- against side scatter (SSC). Then, we determined ARG1 expression in CD33+, CD11b+, CD15+, and CD14+ in circulating MDSCs. If circulating MDSCs in PC are predominantly granulocytic as we have shown in Figure 1, then these cells should express ARG1 but not the monocytic subsets.  Figure 2 shows that ARG1 was only expressed in CD33+, CD11b+, and CD15+. This confirms that ARG1 expression is characteristic for granulocytic MDSCs in pancreatic cancer.

### 3.3. Myeloid Cells Infiltrating Pancreatic Tumour Tissue Are Predominantly Granulocytic MDSC Subset

**Figure 3:**
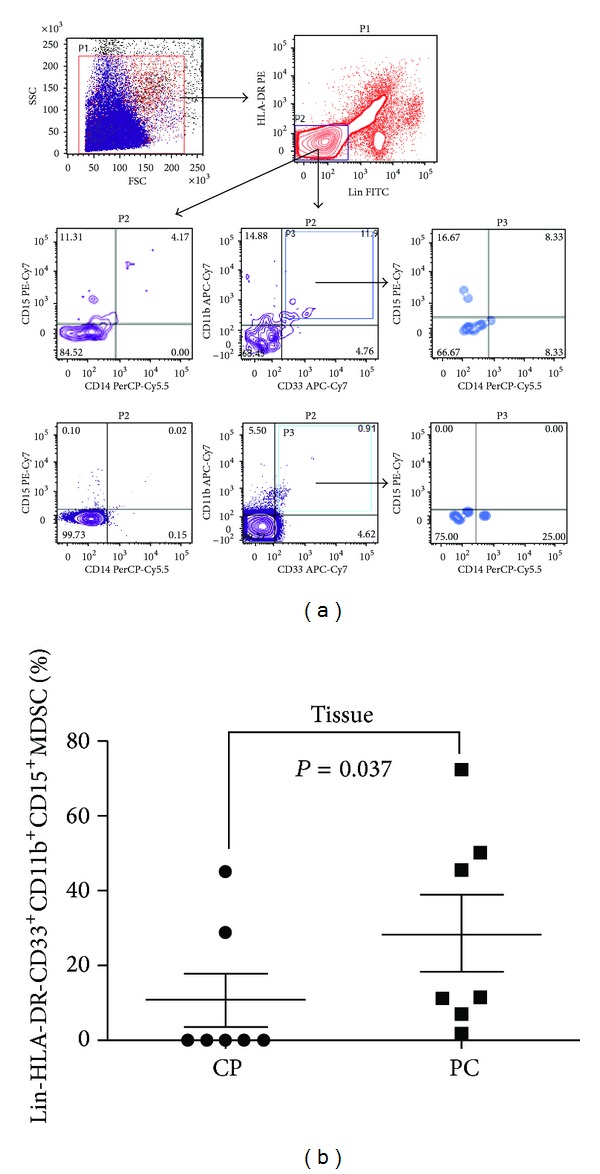
Levels of tumour-infiltrating MDSCs in patients with pancreatic cancer compared to chronic pancreatitis patients. (a) Flow cytometric evaluation of Lin, CD33, CD11b, CD15, and CD14 in tissue samples. An example of representative dot plots is shown for each study sub-group. Gates were set based on controls. Numbers represent the percentages from the original populations gated. P (number) above each FACS plot indicates the population gated that was analysed. The axis of each FACS plot represents the marker analysed. (b) Scatter plot of the percentage of Lin-HLA-DR-CD33+CD11b+CD15+ in the tissue of benign and cancer samples. Bar represents median in each group. CP: chronic pancreatitis; PC: pancreatic cancer.

We examined the pancreatic tumour tissue infiltrate for presence of myeloid cells and we assessed the expression of the same markers used to characterise circulating myeloid cells (Lin, HLA-DR, CD33, CD11b, CD14, and CD15) by flow cytometry in the tumour cell suspensions following enzymatic disaggregation (ED) of fresh surgically resected pancreatic tumour tissues of seven patients with stage I/II PC. Seven benign pancreatic tissues, surgically excised of patients who underwent surgical intervention for the treatment of CP, were analysed in similar manner after ED. The percentage of Lin-HLA-DR-CD33+CD11b+CD15+ MDSC subset in the tissue-infiltrating myeloid cells was markedly increased in the tumour tissue when compared to the matched benign tissue (11.11% versus 0.20%, *P* = 0.037) (Figure 3). Representative flow cytometric plots are shown in Figure 3(a). Although no statistical significant difference was reached in the frequency of infiltrating monocytic Lin-HLA-DR-CD14+ subset in tumour tissues when compared to the benign samples (3.85% versus 0.25%, *P* = 0.209), we observed a 4-fold increase in infiltrating Lin-HLA-DR-CD14+ in comparison to the respective circulating group (3.85% versus 0.885, *P* = 0.072). However, in benign pancreatic tissue samples, we found no increase in the frequency of infiltrating monocytic Lin-HLA-DR-CD14+ when compared to their respective circulating subsets (0.25% versus 63%, *P* = 0.84). The frequency of tumour-infiltrating HLA-DR-CD33+CD11b+CD15+ MDSC subset was markedly higher than that of the monocytic Lin-HLA-DR-CD14+ subset but this did not reach a statistical significance (11.11% versus 3.85%, *P* = 0.073). Therefore, this indicates that these cells are indeed tissue/tumour-infiltrating MDSCs and not just a representation of the blood contained within the tissue samples. Further details of the frequency of the different tissue-infiltrating MDSC subsets that we examined are illustrated in Table 3.

### 3.4. Circulating MDSCs Do Not Correlate with Clinical Cancer Stage

We divided PC patients by clinical cancer stage into an operable group (Stage I/II, *n* = 6) and inoperable group (Stage III/IV, *n* = 18). The percentage of circulating MDSCs in patients with operable tumours was comparable to that of patients with inoperable disease, and this was applicable to both Lin-HLA-DR-CD33+CD11b+CD15+ subset (8.5% versus 8.95%, *P* = 0.45) and monocytic Lin-HLA-DR-CD14+ (1.32% versus 0.63%, *P* = 0.105).

In addition, we also analysed the correlation between the circulating MDSCs and pancreatic tumour size and preoperative serum concentration of CA19-9 and CEA biomarkers. Of the seven cases analysed, six had the record of the tumour size after resection. We observed no correlation between the frequency of circulating MDSCs and tumour size (*P* = 0.86) or with the serum concentration of CA19-9 and CEA cancer biomarkers (*P* = 0.77, 0.86, resp.).

## 4. Discussion

In the present study, we characterised the phenotype and arginase expression in MDSC subsets in pancreatic cancer patients. This is the first study, to date, that examined the frequency and function of different MDSC subsets in blood and tumour tissue of patients with pancreatic cancer in comparison with HDs and benign pancreatic disease in the form of CP. Circulating and tissue-infiltrating granulocytic MDSCs were significantly elevated in the PC patients when compared to respective controls.

Previous work established the presence of granulocytic MDSCs in the circulation of different human cancers including renal, lung cancer (CD11b+CD14-CD15+) [22, 30], breast, colon, and pancreatic cancers (CD15+) [19]. On the other hand, circulating monocytic HLA-DR-CD14+ was found elevated in patients with hepatocellular carcinoma, melanoma, prostate cancer, and multiple myeloma [24–26, 31]. We found that Lin-HLA-DR-CD33+CD11b+CD15+ subset was significantly elevated in the blood of PC patients when compared to HDs. This is concordant with the findings of Gabitass et al. who reported a statistically significant higher frequency of circulating MDSCs in the blood of patients with PC (*n* = 46) compared to HDs (2.1, *P* < 0.001) [32]. However, they only defined MDSC as Lin-HLA-DR-CD33+CD11b+ with no further characterisation of granulocytic or monocytic subsets. In addition, they performed their phenotypical analysis on PBMCs, which could account for the relatively lower levels in comparison to our study. A unique and important finding of our study is that there was no statistical difference in the median of circulating Lin-HLA-DR-CD33+CD11b+CD15+ in the blood of PC patients when compared with CP patients. In another supporting study, Basso and colleagues demonstrated that circulating MDSCs (CD33+CD14-HLA-DR-) were significantly increased (*P* = 0.022) in comparison to HDs, whereas dendritic and cytotoxic T cells were reduced in PC patients [33]. Other groups have described that MDSCs found in the peripheral blood of patients with renal cell carcinoma and PC, derived from PBMC, had the morphology of granulocytes [19, 30]. These MDSCs were CD11b+CD15+, expressed high levels of ARG1, and were negative for macrophage/monocytic marker CD14 [30]. In the present study, we have demonstrated that ARG1 was mainly expressed by granulocytic MDSCs in the circulating of patients with PC. Therefore, ARG1 can be used as a specific marker for the frequency of granulocytic MDSC in cancers. This finding is of significant importance in establishing specific functional MDSC markers for future planned investigations. ARG1 expression levels can be measured by quantitative reverse transcription polymerase chain reaction (QRT-PCR) instead of six colour markers that are needed for the flow cytometric analysis. This is useful for large patient multicentre studies which might be a valuable tool to reduce the variability associated with day-to-day investigations [34]. Collectively, the existing evidence indicates that MDSC expansion in the circulation might favour tumour growth and progression in PC and other human cancers [33].

Our data indicate that MDSCs are expanded not only in cancer progression but also in chronic inflammatory conditions such as CP, and this supports the findings of others [35]. In the last two decades, accumulating evidence has established that longstanding preexisting CP is a strong risk factor for pancreatic cancer with an estimated rate of 5% of PC development [36]. It will be interesting to correlate the levels of MDSCs in the blood of CP patients with the disease burden and with those who develop PC. We found the levels of monocytic MDSCs (HLA-DR-CD14+) to be lower in the blood of PC group in comparison to HDs and CP groups. Although this difference did not reach statistical significance, this may indicate that monocytic MDSCs are expanded in chronic inflammatory conditions including CP. This observation was not described previously but may suggest that granulocytic and monocytic MDSCs have distinct function in humans with chronic inflammation or tumour burden. Therefore, further investigations are required to increase our understanding of the immunosuppressive characteristics of each subset in the circulation.

MDSCs represent a group of immature cells that are morphologically, phenotypically, and functionally heterogeneous and play an important role in cancer immune evasion. This heterogeneity has created a major obstacle to generating specific MDSC markers, and this limited our understanding of their suppressive role in human cancers. However, further identification of the characteristic features of MDSC subsets in different types of human cancers dictated further investigations to redefine MDSCs according to a combination of a new set of markers, such as high levels of CD66b and low levels of CD62L and CD16 [20, 37, 38]. However, the use of these new markers remains controversial due to lack of clinical validation. Therefore, most of the human cancer studies narrowed their characterisations to evaluate the role of the main subpopulations which are granulocytic (Lin-HLA-DR-CD33+CD11b+CD15+) and monocytic (Lin-HLA-DR-CD14+) [39]. Although CD15 is considered as a marker for granulocytes, this phenotype by itself does not discriminate for MDSCs and this was evident in our analysis when we observed no statistical significant difference in the frequency of Lin-HLA-DR-CD15+ across all study groups. In an attempt to further define this subpopulation, we changed the gating strategy to employ two additional markers CD11b and CD33 subsets which yielded significant difference in the frequency of granulocytic MDSC between PC and CP.

Tumour-infiltrating Lin-HLA-DR-CD33+CD11b+CD15+ MDSC subset was significantly expanded in pancreatic tumour in comparison with benign pancreatic tissue. Although our data have shown no significant expansion of monocytic Lin-HLA-DR-CD14+ in the blood of PC patients when compared with HDs, we observed a 4-fold increase in the frequency of the tumour-infiltrating subset. This indicates that both MDSC subpopulations have different functions in different human cancers.

Youn et al. reported, in an analysis of 10 different experimental tumour models, that both MDSC subsets were expanded and the expansion of the granulocytic MDSC population was reported to be greater than that of the monocytic subset [11]. However, their findings suggested that the level of MDSC expansion was not indicative of their suppressive features but rather a representation of functional state within the tumour environment [11]. The impact of MDSCs on cancer could be described as a two-staged effect; the first is an abnormal myelopoiesis and recruitment of MDSCs into the tumour tissue and the second is active MDSC cytokine production and cell-cell interactions within the environment and further progression of cancer [39]. The tumour microenvironment is an important source of specific tumour-associated cytokines and immunosuppressive cells which can modulate the morphology/phenotype of MDSCs. Most of the previously published studies investigated MDSCs in PBMCs or whole blood, and not the tumour tissue, of cancer patients.

We further explored the clinical significance of circulating MDSCs in PC. We found that the percentage of MDSCs in the peripheral blood of PC patients was not correlated with tumour clinical stage, tumour size, or with preoperative cancer biomarkers CA19-9 and CEA. However, these findings can be skewed by the small study sample and a revalidation study with a larger number of patients is mandatory before these conclusions could be drawn. The reports on clinical correlation between circulating/infiltrating MDSC levels and tumour stage and survival are contradicting. In one study, the proportion of MDSCs in CRC tissue was correlated with nodal metastases, distant metastases, and tumour stage suggesting the involvement of MDSCs in cancer development [40]. Others found no such correlation between the percentage of MDSCs and cancer clinical stage [32]. The existing controversies can be explained by the fact that these reports included heterogeneous population of varied cancer types such as breast, lung, melanoma, sarcoma, and gastrointestinal cancers. In addition, the focus of these reports and many other human studies is only directed towards the characterisation of some MDSC subsets in diverse cancer types, which is likely to lead to an inconclusive characterisation of the suppressive features of MDSCs. The processing methods employed for MDSCs characterisation have also varied considerably between studies. Therefore, there is an immense need to validate the prognostic/predictive value of MDSCs in prospective large clinical studies.

Our study is limited by the small number of participants; this was largely due to the exclusion of patients with medical problems (e.g., diabetes mellitus) or concurrent inflammation and sepsis as potential confounding factors. The number of tissue samples was also small due to the small percentage of CP and PC patients who can be treated with surgical resection.

In conclusion, we have evaluated the frequency, phenotype, ARG1 expression, and clinical significance of MDSCs in PC. We demonstrated a significant increase in circulating and tumour-infiltrating MDSC levels and their functional activity in PC. It is likely that the increased frequency of granulocytic MDSCs in human cancers plays an important role in tumour pathogenesis and progression. Although this finding was not shown in our current study, bigger preclinical studies may give a better indication of the diagnostic/prognostic power of MDSCs in PC. Our findings, if incorporated into large prospective validating studies, are of considerable significance for developing new immunotherapeutic strategies via inhibiting and eliminating MDSCs in PC. This work furthers our understanding of the important role of each of these subsets in patients with this cancer and helps to identify new therapeutic targets. In the future, pancreatic cancer treatment might be tailored to target the immunosuppressive pathways used by MDSCs.

## Figures and Tables

**Table 1:** Characteristic features of study subpopulations.

	PC	CP	HDs
Number	*n* = 24	*n* = 12	*n* = 16
Age (median)	64.5 (41–85)*	49 (23–72)*	51 (24–89)*
Gender (male : female)	13 : 11	5 : 7	7 : 9
TNM stage			
I	0	—	—
II	6	—	—
III	1	—	—
IV	17	—	—
Tumour size (cm)	3.2 (1.9–6.1)*		
Preoperative CA19-9 (0–37 U/mL)	399 (77–1230)*	54	—
Preoperative CEA (<2.5 ng/mL)	5 (5–13)*	—	—
Tumour site			
Head of pancreas	18	—	—
Body of pancreas	2	—	—
Tail of pancreas	4	—	—
Histological grade			
Well/moderate	6	—	—
Poor/undifferentiated	18	—	—

PC: pancreatic cancer; CP: chronic pancreatitis; HDs: healthy donors; CA19-9: cancer antigen 19-9; CEA: carcinoembryonic antigen.*Data shown represent median (range).

**Table 2:** Frequency of MDSC subsets in the peripheral blood of healthy donors, chronic pancreatitis, and pancreatic cancer patients.

	PC	CP	HDs	*P* value
Lin-HLA-DR-	2.7 (0.7–17.5)*	1.9 (1–90.7)*	4.3 (1.0–14.6)*	A = 0.14, B = 0.59, C = 0.79
Lin-HLA-DR-CD33+11b+	8.1 (1.4–27.6)*	11.8 (0.5–34.2)*	8.7 (0–19.2)*	A = 0.6, B = 0.85, C = 0.89
Lin-HLA-DR-CD33+11b+CD15+	8.8 (0–91.3)*	8.9 (0–33.7)*	1.4 (0–14.5)*	**A = 0.0003**, B = 0.65, C = 0.13
Lin-HLA-DR-CD14+	0.8 (0.2–6.6)*	1.6 (0.4–6.3)*	1.3 (0.16–3.5)*	A = 0.47, B = 0.43, C = 0.63
HLA-DR-/^low^CD14+	0.9 (0.2–7.5)*	2.2 (0.2–37.1)*	1.8 (0.6–6.7)*	A = 0.41, B = 0.83, C = 0.79
HLA-DR-/^Low^CD33+	10.5 (1.9–31.2)*	14.8 (0.4–93.1)*	14.2 (1.4–23.9)*	A = 0.69, B = 0.818, C = 0.89

PC: pancreatic cancer; CP: chronic pancreatitis; HDs: healthy donors. *P* value: A = (PC versus HDs); B = (PC versus CP); C = (CP versus HDs).*Data shown represent median (range). The bold values refer to statistically significant *P* values.

**Table 3:** Frequency of tissue infiltrating MDSC subsets in benign and malignant pancreatic tissue.

	PC	CP	*P* value
Lin-HLA-DR-	57.3 (9.4–90.9)*	91.82 (10.6–95.8)*	0.07
Lin-HLA-DR-CD33+11b+	2.9 (1.4–27.6)*	0.35 (0.03–13.7)*	0.48
Lin-HLA-DR-CD33+11b+CD15+	11.1 (0.5–72.3)*	0.2 (0–45.1)*	**0.037**
Lin-HLA-DR-CD14+	2.5 (0.19–5.07)*	0.3 (0.1–17.5)*	0.66
HLA-DR-/^low^CD14+	2.7 (0.5–5.1)*	2.3 (0–14.1)*	0.62
HLA-DR-/^Low^CD33+	6.9 (0.82–25.5)*	11.5 (0.31–13.6)*	0.82

PC: pancreatic cancer; CP: chronic pancreatitis; HDs: healthy donors.*Data shown represent median (range). The bold values refer to statistically significant *P* values.
